# Numerical approximation for algorithmic tangent moduli for nonlinear viscoelastic model with CSDA method

**DOI:** 10.1038/s41598-024-65441-2

**Published:** 2024-08-07

**Authors:** Xinggui Fan, Jinsheng Xu, Xiong Chen

**Affiliations:** Nanjing University of Science and Technology, Xiaolingwei 200#, Xuanwu District, Nanjing, Jiangsu China

**Keywords:** Viscoelastic model, Algorithmic tangent moduli, CSDA, Finite element method, Mechanical engineering, Computational methods

## Abstract

This work revisits the notion of complex step derivative approximation (CSDA) and presents its use in constitutive model of a class of nonlinear viscoelastic materials. The effectiveness of a CSDA is evaluated by putting it through a series of straightforward examples. After that, the idea of the CSDA is put to use in order to carry out a numerical evaluation of the algorithmic tangent moduli of a viscoelastic constitutive model. The performance of the constitutive models is evaluated through the use of three different numerical tests, and the results are compared to those that were achieved by the application of an analytical method. In comparison to other numerical differentiation techniques, It has been found that the CSDA scheme is the most computationally efficient and robust method of numerical differentiation, regardless of the size of the finite difference interval.

## Introduction

In finite element simulation, the constitutive model is an essential aspect that provides stress and algorithmic tangent matrix update for the main program. Despite the fact that commercial finite element modeling software includes a large amount of material models, it is challenging to address the growing number of engineering challenges that require more complex constitutive models. Given this, the commercial finite element simulation program might be provided with a secondary developer interface for expanding its material library. The ABAQUS program offers the UMAT secondary development interface to fulfill this function, which requires Cauchy stress and algorithmic tangent matrix update. When evaluating a algorithmic tangent matrix, the approach that is typically utilized is one in which the analytic derivative of material models is deduced. Due to the increasing mathematical complexity of constitutive models, it is becoming increasingly difficult or nearly impossible to deduce an analytic algorithmic tangent matrix of constitutive models. This is because constitutive model complexity is increasing (such as finite plasticity, hyperelasticity, thermo-elasticity, anisotropic magneto-mechanical model or viscoelasticity based on fractional calculus^1–4^). In situations like these, numerical differentiation could be an effective alternate method to apply when attempting to analyze the algorithmic tangent matrix. Miehe^5^ was the first one who deduced a numerical differentiation procedure for the algorithmic tangent matrix in large-strain inelasticity. The numerical differentiation procedure is a perturbation technique based on finite difference approximation and material-independent. In later years, the approach that Miehe had suggested was utilized in the numerical evaluation of a number of other constitutive models ^6–9^. Based on Miehe’s work, A numerical approximation for the algorithmic tangent matrix was derived by Sun^10^. This approximation was based on the Jaumann rate of Kirchhoff stress for the hyperelastic material model. Sun’s method was then used to evaluate the algorithmic tangent matrix of material models such as the anisotropic hyperelastic model^11,12^, crystal plasticity^13^, a model for shape-memory polymers^14^, and a model of tissue fatigue^15,16^. To improve computational accuracy, Perez-Foguet et al.^6,17^ proposed a central difference method (CDM, second-order accuracy) instead of the forward difference method (FDM, first-order accuracy) to evaluate the algorithmic tangent matrix for small strain elastoplastic problems. Both the FDM and CDM, however, cause subtractive cancellation errors, which are the result of performing these calculations on a computer with finite precision. In terms of the truncation errors, it is desirable to reduce the size of the finite difference interval as far as possible, while subtractive cancellation errors become dominant as the size of the finite difference interval decreases. Hence, the optimum size of the finite difference interval is one that strikes a compromise between the errors caused by truncation and those caused by subtractive cancellation. A numerical differential method based on the idea of CSDA proposed by Lyness^18^ was extended to evaluate the algorithmic tangent matrix ^19–23^, which eliminates the effect of subtractive cancellation errors. The CSDA does not require a subtraction operation, and as a result, it offers a larger range of perturbation while still ensuring high precision approximation. This represents a significant improvement over both the FDM and the CDM. When dealing with approximations of higher-order derivatives, the CSDA is susceptible to subtractive cancellation mistakes as well. A numerical technique that is based on the concept of hyper-dual numbers (HDNs) that was proposed by Fike^24^ was recently used to assess the algorithmic tangent matrix. This scheme was recently adopted since it was inspired by the CSDA. The HDNs have more than one non-real unit number, which makes it easier to evaluate second-order derivatives (or higher order derivatives) and eliminates the possibility of errors caused by subtractive cancellation. Researchers have developed a variety of numerical approximation approaches of the algorithmic tangent matrix for a wide range of constitutive models by utilizing the HDSD, also known as the hyper-dual step derivative ^25^.

In the current investigation, it was decided that a numerical approximation scheme of the algorithmic tangent matrix for a class of nonlinear viscoelastic model should be developed. This decision was made after it was determined that such a scheme would be beneficial. In spite of the fact that the HDSD is superior to the CSDA when it comes to evaluating derivatives of a higher order (or higher orders), for the purpose of this research, it is only necessary to evaluate the derivative of the second Piola–Kirchhoff stress with respect to the right Cauchy-Green strain tensor. The CSDA will evaluate a numerical approximation algorithmic tangent matrix and then compare it to an analytic algorithmic tangent matrix. This will be done in the interest of keeping things as straightforward as possible. The remaining parts of this work are structured as described below. In the second section, we derive a recursive updating method for analytic Cauchy stress as well as a spatially algorithmic tangent matrix. In "Numerical approximations of algorithmic tangent moduli for the class of a viscoelastic model", we derive an update implementation for numerically algorithmic spatial tangent matrices that uses a recursive algorithm. There is a comparison of the two techniques discussed earlier with the help of some numerical examples in "Numerical testing". In "Conclusions", the conclusions are discussed.

## Analytical algorithmic tangent moduli for the class of a viscoelastic model

### Finite nonlinear viscoelastic model

**Figure 1:**
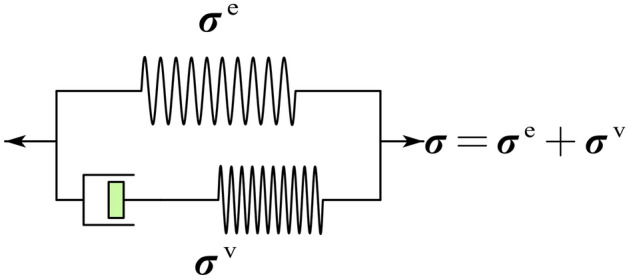
Rheological model of standard solid.

For rubber-like materials, a rheological model of the standard solid is usually employed to characterize its mechanical response. A schematic of this model is shown below (Fig. 1).

The one-dimensional infinitesimal linear viscoelastic standard solid model is expressed in convolution form as

1 $$ \sigma \left( t \right) = \int_{ - \infty }^{t} {G\left( {t - s} \right)\dot{\varepsilon }{\text{d}}s} $$

where

2 $$ G\left( t \right) = \gamma_{\infty } + \sum\limits_{i = 1}^{N} {\gamma_{i} \exp \left( { - t/\theta_{i} } \right)} $$

For a finite deformation, Simo^5^ generalized the infinitesimal linear viscoelastic standard solid model to a finite nonlinear case

3 $$ \begin{gathered} {\varvec{S}}\left( t \right) = J\left( t \right)U^{ \circ } \prime \left( {J\left( t \right)} \right){\varvec{C}}\left( t \right)^{ - 1} + \gamma_{\infty } J\left( t \right)^{ - 2/3} {\text{DEV}}\left\{ {2\partial_{{\overline{{\varvec{C}}} }} \overline{W}^{ \circ } \left( {\overline{{\varvec{C}}} \left( t \right)} \right)} \right\}{\mkern 1mu} {\mkern 1mu} \\ + \sum\limits_{i = 1}^{N} {J\left( t \right)^{ - 2/3} \gamma_{i} \int_{ - \infty }^{t} {\exp \left( { - \left( {t - s} \right)/\theta_{i} } \right)\frac{{\text{d}}}{{{\text{d}}s}}\left( {{\text{DEV}}\left\{ {2\partial_{{\overline{{\varvec{C}}} }} \overline{W}^{ \circ } \left( {\overline{{\varvec{C}}} \left( s \right)} \right)} \right\}} \right){\text{d}}s} } \\ \end{gathered} $$

Here, the stored-energy function is decomposed into a volumetric part $U^{ \circ } \left( J \right)$ and a volumetric-preserving part $\overline{W}^{ \circ } \left( {\overline{{\varvec{C}}} } \right)$. The deformation gradient ***F*** is also decomposed into a volumetric part $J{\varvec{1}}$ and a volumetric-preserving part $\overline{{\varvec{F}}}$, namely

4 $$ \overline{{\varvec{F}}} = J^{ - 1/3} {\varvec{F}} $$

where $J = \det \left[ {\varvec{F}} \right]$ is the Jacobian determinant of the deformation gradient ***F*** and $\det \left[ {\overline{{\varvec{F}}} } \right] = 1$. The deviatoric right Cauchy-Green strain tensor is given by

5 $$ \overline{{\varvec{C}}} = \overline{{\varvec{F}}}^{{\text{T}}} \overline{{\varvec{F}}} $$

${\varvec{S}}\left( t \right)$ is the second Piola–Kirchhoff stress, which is also decomposed into volumertric and volume-preserving parts. It is assumed that its volumetric part is elastic, where

$$ U^{ \circ } \prime \left( J \right) = \frac{{{\text{d}}U^{ \circ } \left( J \right)}}{{{\text{d}}J}} $$

$$ {\text{DEV[}} \cdot {]} = ( \cdot ) - \frac{1}{3}\left[ {( \cdot ):{\varvec{C}}} \right]{\varvec{C}}^{ - 1} $$

Thus, the Kirchhoff stress ${{\varvec{\uptau}}}\left( t \right)$ is written as

6 $$ \begin{gathered} {{\varvec{\uptau}}}\left( t \right) = {\varvec{FSF}}^{{\text{T}}} = J\left( t \right)U^{ \circ } \prime \left( {J\left( t \right)} \right){\varvec{1}} + \gamma_{\infty } {\text{dev}}\left[ {2\overline{{\varvec{F}}} \left( t \right)\partial_{{\overline{{\varvec{C}}} }} \overline{W}^{ \circ } \left[ {\overline{{\varvec{C}}} \left( t \right)} \right]\overline{{\varvec{F}}} \left( t \right)^{{\text{T}}} } \right] + \\ \sum\limits_{i = 1}^{N} {\gamma_{i} \int_{ - \infty }^{t} {\exp \left( { - \left( {t - s} \right)/\theta_{i} } \right)\frac{{\text{d}}}{{{\text{d}}s}}\left( {{\text{dev}}\left\{ {2\overline{{\varvec{F}}} \left( s \right)\partial_{{\overline{{\varvec{C}}} }} \overline{W}^{ \circ } \left[ {\overline{{\varvec{C}}} \left( s \right)} \right]\overline{{\varvec{F}}} \left( s \right)^{{\text{T}}} } \right\}} \right){\text{d}}s} } \\ \end{gathered} $$

where

$$ {\text{dev}}\left[ \cdot \right] = \left( \cdot \right) - \frac{1}{3}\left[ {\left( \cdot \right):{\varvec{1}}} \right]{\varvec{1}} $$

and **1** is the second-order identity tensor.

### Implementation of integration algorithms for a nonlinear viscoelastic model

#### Cauchy stress ${{\varvec{\upsigma}}}_{n + 1}$

It is important to derive the time-discrete form of the proposed model in order to implement it in finite element software (for example, ABAQUS) in the form of a user-defined material subroutine. This will allow the model to be implemented in this study. First, the time interval [0,* t*] is divided into sub-increments. Further, lettingthe so-called internal variable is then defined by

$$ \left[ {0,t} \right] = \bigcup {\left[ {t_{n} ,t_{n + 1} } \right]} ,{\kern 1pt} {\kern 1pt} {\kern 1pt} {\kern 1pt} {\kern 1pt} {\kern 1pt} {\kern 1pt} {\kern 1pt} {\kern 1pt} t_{n + 1} = t_{n} + {\Delta }t $$

7 $$ {\varvec{H}}^{\left( i \right)} \left( t \right) = \int_{{T_{0} }}^{t} {\exp \left( { - \left( {t - s} \right)/\theta_{i} } \right)\frac{{\text{d}}}{{{\text{d}}s}}\left( {{\text{DEV}}\left[ {2\partial_{{\overline{{\varvec{C}}} }} \overline{W}^{ \circ } \left[ {\overline{{\varvec{C}}} \left( s \right)} \right]} \right]} \right){\text{d}}s} $$

For $t_{n + 1} = t_{n} + {\Delta }t$, according to Eq. (7), using the additive property of the integral, for the sake of convenience, we show the following quantities related to time $t_{n}$ and with *n* and *n* + 1, respectively, which yield

8 $$ {\varvec{H}}_{n + 1}^{\left( i \right)} = \exp \left( { - {\Delta }t/\theta_{i} } \right){\varvec{H}}_{n}^{\left( i \right)} + \int_{{t_{n} }}^{{t_{n + 1} }} {\exp \left( { - \left( {t_{n + 1} - s} \right)/\theta_{i} } \right)\frac{{\text{d}}}{{{\text{d}}s}}\left( {{\text{DEV}}\left[ {2\partial_{{\overline{{\varvec{C}}} }} \overline{W}^{ \circ } \left[ {\overline{{\varvec{C}}} \left( s \right)} \right]} \right]} \right){\text{d}}s} $$

For the second term on the right hand side of Eq. (8), the midpoint rule is used, yielding

9 $$ \begin{gathered} \int_{{t_{n} }}^{{t_{n + 1} }} {\exp \left( { - \left( {t_{n + 1} - s} \right)/\theta_{i} } \right)\frac{{\text{d}}}{{{\text{d}}s}}\left( {{\text{DEV}}\left[ {2\partial_{{\overline{{\varvec{C}}} }} \overline{W}^{ \circ } \left[ {\overline{{\varvec{C}}} \left( s \right)} \right]} \right]} \right){\text{d}}s} \hfill \\ \approx \exp \left( { - \left( {t_{n + 1} - s} \right)/\theta_{i} } \right)\frac{{\text{d}}}{{{\text{d}}s}}\left( {{\text{DEV}}\left[ {2\partial_{{\overline{{\varvec{C}}} }} \overline{W}^{ \circ } \left[ {\overline{{\varvec{C}}} \left( s \right)} \right]} \right]} \right)\left| {_{{s = \frac{{t_{n} + t_{n + 1} }}{2}}} } \right.{\Delta }t \hfill \\ = \exp \left( { - {\Delta }t/\left( {2\theta_{i} } \right)} \right)\left( {\widetilde{{\varvec{S}}}_{n + 1} - \widetilde{{\varvec{S}}}_{n} } \right) \hfill \\ \end{gathered} $$

where

$$ \widetilde{{\varvec{S}}}_{n + 1} = {\text{DEV}}\left[ {2\partial_{{\overline{{\varvec{C}}} }} \overline{W}^{ \circ } \left[ {\overline{{\varvec{C}}}_{n + 1} } \right]} \right] $$

$$ \widetilde{{\varvec{S}}}_{n} = {\text{DEV}}\left[ {2\partial_{{\overline{{\varvec{C}}} }} \overline{W}^{ \circ } \left[ {\overline{{\varvec{C}}}_{n} } \right]} \right] $$

Combining Eqs. (8) and (9), the approximation of ${\varvec{H}}_{n + 1}^{\left( i \right)}$ is obtained as follows

10 $$ {\varvec{H}}_{n + 1}^{\left( i \right)} = \exp \left( { - {\Delta }t/\theta_{i} } \right){\varvec{H}}_{n}^{\left( i \right)} + \exp \left( { - {\Delta }t/\left( {2\theta_{i} } \right)} \right)\left( {\widetilde{{\varvec{S}}}_{n + 1} - \widetilde{{\varvec{S}}}_{n} } \right) $$

With these formulas in hand, ${\varvec{S}}_{n + 1}$ can be written as

11 $$ {\varvec{S}}_{n + 1} = J_{n + 1} U^{ \circ } \prime \left( {J_{n + 1} } \right){\varvec{C}}_{n + 1}^{ - 1} + \gamma_{\infty } \overline{{\varvec{S}}}_{n + 1}^{ \circ } + \sum\limits_{i = 1}^{N} {J_{n + 1}^{ - 2/3} \gamma_{i} {\text{DEV}}_{n + 1} \left[ {{\varvec{H}}_{n + 1}^{\left( i \right)} } \right]} $$

where

12 $$ \overline{{\varvec{S}}}_{n + 1}^{ \circ } = J_{n + 1}^{ - 2/3} {\text{DEV}}_{n + 1} \left[ {2\partial_{{\overline{{\varvec{C}}} }} \overline{W}^{ \circ } \left[ {\overline{{\varvec{C}}}_{n + 1} } \right]} \right] $$

Combining Eqs. (10) and (11), the Kirchhoff stress is given by

13 $$ {{\varvec{\uptau}}}_{n + 1} = J_{n + 1} U^{ \circ } \prime \left( {J_{n + 1} } \right){\varvec{1}} + g^{ * } \left( {{\Delta }t} \right)\overline{{{\varvec{\uptau}}}}_{n + 1} + \overline{{\varvec{h}}}_{n} $$

where

$$ g^{ * } \left( {{\Delta }t} \right) = \gamma_{\infty } + \sum\limits_{i = 1}^{N} {\gamma_{i} \exp \left( { - {\Delta }t/\left( {2\theta_{i} } \right)} \right)} $$

$$ \overline{{{\varvec{\uptau}}}}_{n + 1}^{ \circ } = {\text{dev}}\left\{ {2\overline{{\varvec{F}}}_{n + 1} \partial_{{\overline{{\varvec{C}}} }} \overline{W}^{ \circ } \left[ {\overline{{\varvec{C}}}_{n + 1} } \right]\overline{{\varvec{F}}}_{n + 1}^{{\text{T}}} } \right\} $$

$$ \overline{{\varvec{h}}}_{n} = \sum\limits_{i = 1}^{N} {\gamma_{i} {\text{dev}}\left\{ {\overline{{\varvec{F}}}_{n + 1} \widetilde{{\varvec{H}}}_{n}^{\left( i \right)} \overline{{\varvec{F}}}_{n + 1}^{{\text{T}}} } \right\}} $$

$$ \widetilde{{\varvec{H}}}_{n}^{\left( i \right)} = \exp \left( { - {\Delta }t/\theta_{i} } \right){\varvec{H}}_{n}^{\left( i \right)} - \exp \left( { - \Delta t/\left( {2\theta_{i} } \right)} \right)\widetilde{{\varvec{S}}}_{n}^{ \circ } $$

$$ \widetilde{{\varvec{S}}}_{n + 1}^{ \circ } = \overline{{\varvec{F}}}_{n + 1}^{ - 1} \overline{{{\varvec{\uptau}}}}_{n + 1}^{ \circ } \overline{{\varvec{F}}}_{n + 1}^{{ - {\text{T}}}} $$

Dividing ${{\varvec{\uptau}}}_{n + 1}$ by $J_{n + 1}$, the Cauchy stress is obtained as follows:

14 $$ \sigma_{ij}^{n + 1} = \frac{1}{{J_{n + 1} }}\tau_{ij}^{n + 1} $$

#### Algorithmic spatial tangent moduli ${\varvec{c}}_{n + 1}$

Differentiation of the second Piola–Kirchhoff stress tensor ${\varvec{S}}_{n + 1}$ with respect to ${\varvec{C}}_{n + 1}$ yields the material tangent moduli, namely,

15 $$ {\varvec{C}}_{n + 1} = 2\partial_{{{\varvec{C}}_{n + 1} }} {\varvec{S}}_{n + 1} $$

The hyperelastic second Piola–Kirchhoff stress can be written as

16 $$ {\varvec{S}} = JU^{\prime}{\varvec{C}}^{ - 1} + \overline{{\varvec{S}}} $$

where $\overline{{\varvec{S}}}$ is the deviatoric part of the second Piola–Kirchhoff stress.

Differentiating Eqs. (16)with respect to ${\varvec{C}}_{n + 1}$, we obtain

17 $$ {\varvec{C}}_{n + 1}^{ \circ } = 4U^{ \circ ^{\prime\prime}} \left( {J_{n + 1} } \right)\frac{{\partial J_{n + 1} }}{{\partial {\varvec{C}}_{n + 1} }} \otimes \frac{{\partial J_{n + 1} }}{{\partial {\varvec{C}}_{n + 1} }} + U^{ \circ } \prime \left( {J_{n + 1} } \right)\frac{{\partial^{2} J_{n + 1} }}{{\partial {\varvec{C}}_{n + 1} \partial {\varvec{C}}_{n + 1} }} + \overline{{\varvec{C}}}_{n + 1}^{ \circ } $$

Similarly, the following are set

18 $$ \overline{{\varvec{C}}}_{n + 1} = 2\partial_{{{\varvec{C}}_{n + 1} }} \overline{{\varvec{S}}}_{n + 1} $$

19 $$ \overline{{\varvec{S}}}_{n + 1} = g^{ * } \left( {\Delta t} \right)\overline{{\varvec{S}}}_{n + 1}^{ \circ } + \sum\limits_{i = 1}^{N} {\gamma_{i} J_{n + 1}^{ - 2/3} {\text{DEV}}_{n + 1} \left[ {\widetilde{{\varvec{H}}}_{n}^{\left( i \right)} } \right]} $$

20 $$ \overline{{\varvec{S}}}_{n + 1} = g^{ * } \left( {{\Delta }t} \right)\overline{{\varvec{S}}}_{n + 1}^{ \circ } + \sum\limits_{i = 1}^{N} {\gamma_{i} J_{n + 1}^{ - 2/3} {\text{DEV}}_{n + 1} \left[ {\widetilde{{\varvec{H}}}_{n}^{\left( i \right)} } \right]} $$

21 $$ \begin{gathered} \overline{{\varvec{C}}}_{n + 1} = g^{ * } \left( {\Delta t_{n} } \right)\overline{{\varvec{C}}}_{n + 1}^{ \circ } + \sum\limits_{i = 1}^{N} {\left[ {\gamma_{i} J_{n + 1}^{ - 2/3} \left\{ { - \frac{2}{3}{\text{DEV}}_{n + 1} \left[ {\widetilde{{\varvec{H}}}_{n}^{\left( i \right)} } \right] \otimes {\varvec{C}}_{n + 1}^{ - 1} } \right.} \right.} {\kern 1pt} {\kern 1pt} \\ - \frac{2}{3}{\varvec{C}}_{n + 1}^{ - 1} \otimes {\text{DEV}}_{n + 1} \left[ {\widetilde{{\varvec{H}}}_{n}^{\left( i \right)} } \right]{\kern 1pt} {\kern 1pt} \left. { + \frac{2}{3}\left( {\widetilde{{\varvec{H}}}_{n}^{\left( i \right)} :{\varvec{C}}_{n + 1} } \right)\left( {I_{{\overline{{\varvec{C}}}_{n + 1} }} - \frac{1}{3}{\varvec{C}}_{n + 1}^{ - 1} \otimes {\varvec{C}}_{n + 1}^{ - 1} } \right)} \right] \\ \end{gathered} $$

where

22 $$ \begin{gathered} \overline{{\varvec{C}}}_{{{\text{DEV}}}}^{ \circ } = 4J^{ - 4/3} \left[ {\partial_{{\overline{{\varvec{C}}} \overline{{\varvec{C}}} }} \overline{W}^{ \circ } + \frac{1}{9}\left( {{\varvec{C}}:\partial_{{\overline{{\varvec{C}}} \overline{{\varvec{C}}} }}^{2} \overline{W}^{ \circ } :{\varvec{C}}} \right){\varvec{C}}^{ - 1} \otimes {\varvec{C}}^{ - 1} } \right. \\ \left. { - \frac{1}{3}{\varvec{C}}^{ - 1} \otimes \left( {\partial_{{\overline{{\varvec{C}}} \overline{{\varvec{C}}} }}^{2} \overline{W}^{ \circ } :{\varvec{C}}} \right) - \frac{1}{3}\left( {\partial_{{\overline{{\varvec{C}}} \overline{{\varvec{C}}} }}^{2} \overline{W}^{ \circ } :{\varvec{C}}} \right) \otimes {\varvec{C}}^{ - 1} } \right] \\ \end{gathered} $$

The spatial algorithmic tangent moduli are obtained by pushing forward the material algorithmic tangent moduli to the current configuration, namely

23 $$ {\varvec{c}}_{n + 1} = J_{n + 1}^{2} U^{{ \circ^{\prime \prime } }} \left( {J_{n + 1} } \right){\varvec{1}} \otimes {\varvec{1}} + J_{n + 1} U^{ \circ \prime } \left( {J_{n + 1} } \right)\left( {{\varvec{1}} \otimes {\varvec{1}} - 2{\varvec{I}}} \right) + \overline{{\varvec{c}}}_{n + 1} $$

24 $$ c_{ijkl} = F_{iK} F_{jL} F_{kM} F_{lN} C_{KLMN} $$

25 $$ {\varvec{c}}_{n + 1} = J_{n + 1}^{2} U^{{ \circ^{\prime \prime } }} \left( {J_{n + 1} } \right){\varvec{1}} \otimes {\varvec{1}} + J_{n + 1} U^{ \circ \prime } \left( {J_{n + 1} } \right)\left( {{\varvec{1}} \otimes {\varvec{1}} - 2{\varvec{I}}} \right) + \overline{{\varvec{c}}}_{n + 1} $$

where

26 $$ \begin{gathered} \overline{{\varvec{c}}}_{ijkl}^{n + 1} = g^{ * } \left( {{\Delta }t_{n} } \right)\overline{{\varvec{c}}}_{n + 1}^{ \circ } \left| {_{ijkl} } \right. + \sum\limits_{i = 1}^{N} {\gamma_{i} \left[ {\left\{ { - \frac{2}{3}{\text{dev}}\left[ {\overline{{\varvec{F}}}_{n + 1} \widetilde{{\varvec{H}}}_{n}^{\left( i \right)} \overline{{\varvec{F}}}_{n + 1}^{{\text{T}}} } \right]_{ij} \otimes \delta_{kl} } \right.} \right.} \hfill \\ \left. { - \frac{2}{3}\delta_{ij} \otimes {\text{dev}}\left[ {\overline{{\varvec{F}}}_{n + 1} \widetilde{{\varvec{H}}}_{n}^{\left( i \right)} \overline{{\varvec{F}}}_{n + 1}^{{\text{T}}} } \right]\left| {_{kl} } \right. + \frac{2}{3}{\text{tr}}\left( {\overline{{\varvec{F}}}_{n + 1} \widetilde{{\varvec{H}}}_{n}^{\left( i \right)} \overline{{\varvec{F}}}_{n + 1}^{{\text{T}}} } \right)\left( {\frac{1}{2}\left( {\delta_{ik} \delta_{jl} + \delta_{il} \delta_{jk} } \right) - \frac{1}{3}\delta_{ij} \delta_{kl} } \right)} \right] \hfill \\ \end{gathered} $$

where **I** in Eq. (25) is the fourth-order identity tensor.

In ABAQUS, the spatial algorithmic tangent moduli ${\varvec{c}}_{n + 1}^{{{\text{ANA}} - {\text{MZ}} - {\text{J}}}}$ is defined in terms of the Cauchy stress tensor, namely^26^,

27 $$ \begin{gathered} {\varvec{c}}_{n + 1}^{{{\text{ANA}} - {\text{MZ}} - {\text{J}}}} = \frac{1}{{J_{n + 1} }}\left( {{\varvec{c}}_{n + 1} + \left( {\delta^{ik} \tau_{n + 1}^{jl} + \delta^{jl} \tau_{n + 1}^{ik} + \delta^{il} \tau_{n + 1}^{jk} + \delta^{jk} \tau_{n + 1}^{il} } \right)} \right. \\ \left. {{\varvec{e}}_{i} \otimes {\varvec{e}}_{j} \otimes {\varvec{e}}_{k} \otimes {\varvec{e}}_{l} } \right) \\ \end{gathered} $$

## Numerical approximations of algorithmic tangent moduli for the class of a viscoelastic model

Linearized Jaumann rate of Kirchhoff stress tensor is the basis for the numerical approximation of algorithmic tangent moduli, namely

28 $$ {\Delta }{{\varvec{\uptau}}} - {\Delta }{\varvec{W\tau }} - {{\varvec{\uptau}}}{\Delta }{\varvec{W}}^{{\text{T}}} = {\varvec{c}}:{\Delta }{\varvec{D}} $$

where ${\Delta }{{\varvec{\uptau}}}$, ${\Delta }{\varvec{W}}$, ${\Delta }{\varvec{D}}$ are the increment in the Kirchhoff stress, spin tensor, rate of the deformation tensor, respectively. ***c*** is the spatial algorithmic tangent matrix.

Considering the Taylor series expansion for an analytical real function *f*(*x*), we obtain

29 $$ f\left( {x + h} \right) = f\left( x \right) + hf\prime \left( x \right) + \frac{{h^{2} }}{2}f^{\prime\prime}\left( x \right) + {\mathcal{O}}\left( {h^{2} } \right) $$

The so-called forward-difference scheme gives

30 $$ f^{\prime}_{{{\text{FD}}}} \approx \frac{{f\left( {x + h} \right) - f\left( x \right)}}{h} $$

To eliminate the effect of the subtractive cancellation errors that the FDM scheme causes, *h* is replaced by i*h* in Eq. (29), yielding

31 $$ f\left( {x + {\text{i}}h} \right) = f\left( x \right) + {\text{i}}hf\prime \left( x \right) - \frac{{h^{2} }}{2}f^{\prime\prime}\left( x \right) + {\mathcal{O}}\left( {h^{2} } \right) $$

It can be seen that $f\prime \left( x \right)$ could be obtained by taking the imaginary part of Eq. (31):

32 $$ f_{{{\text{CSDA}}}}^{\prime } \left( x \right) \approx \frac{{{\text{Im}}\left[ {f\left( {x + {\text{i}}h} \right)} \right]}}{h} $$

where ${\text{Im}}\left[ \cdot \right]$ denotes the operation of taking the imaginary part of complex functions. This is the so-called CSDA scheme that avoids subtraction cancellation errors. Generalizing the CSDA to the tensor function of second order, the increment in the Kirchhoff stress ${\Delta }{{\varvec{\uptau}}}$ is approximated by the CSDA scheme and is given as

33 $$ {\Delta }{{\varvec{\uptau}}}^{{\left( {ij} \right)}} \approx {\text{Im}}\left[ {{{\varvec{\uptau}}}\left( {\widehat{{\varvec{F}}}^{{\left( {ij} \right)}} } \right)} \right] $$

where

34 $$ \widehat{{\varvec{F}}}^{{\left( {ij} \right)}} = {\varvec{F}} + {\text{i}}\Delta {\varvec{F}}^{{\left( {ij} \right)}} $$

Following Miehe^5^, the increment in the deformation gradient ${\Delta }{\varvec{F}}^{{\left( {ij} \right)}}$ is defined as

35 $$ {\Delta }{\varvec{F}}^{{\left( {ij} \right)}} = \frac{h}{2}\left( {{\varvec{e}}_{i} \otimes {\varvec{e}}_{j} {\varvec{F}} + {\varvec{e}}_{j} \otimes {\varvec{e}}_{i} {\varvec{F}}} \right) $$

There, ${\Delta }{\varvec{W}}^{{\left( {ij} \right)}}$ and ${\Delta }{\varvec{D}}^{{\left( {ij} \right)}}$ can be written, respectively, as

36 $$ {\Delta }{\varvec{W}} = \frac{1}{2}\left( {{\Delta }{\varvec{FF}}^{ - 1} - \left( {{\Delta }{\varvec{FF}}^{ - 1} } \right)^{{\text{T}}} } \right) = {0} $$

37 $$ \begin{gathered} {\Delta }{\varvec{D}}^{{\left( {ij} \right)}} = \frac{1}{2}\left( {{\Delta }{\varvec{F}}^{{\left( {ij} \right)}} {\varvec{F}}^{ - 1} + \left( {{\Delta }{\varvec{F}}^{{\left( {ij} \right)}} {\varvec{F}}^{ - 1} } \right)^{{\text{T}}} } \right) \\ = \frac{h}{2}\left( {{\varvec{e}}_{i} \otimes {\varvec{e}}_{j} + {\varvec{e}}_{j} \otimes {\varvec{e}}_{i} } \right) \\ \end{gathered} $$

By inserting Eqs. (36), and (37) into Eq. (28), we obtain

38 $$ {\Delta }{{\varvec{\uptau}}}^{{\left( {ij} \right)}} \approx {\varvec{c}}^{{{\tau J}\left( {ij} \right)}} :\frac{h}{2}\left( {{\varvec{e}}_{i} \otimes {\varvec{e}}_{j} + {\varvec{e}}_{j} \otimes {\varvec{e}}_{i} } \right) $$

where ${\varvec{c}}^{{{\tau J}\left( {ij} \right)}}$ is obtained by the perturbation of ${\Delta }{\varvec{F}}^{{\left( {ij} \right)}}$.

Applying the minor symmetry characteristics of the tangent moduli yields the following results:

39 $$ \left( {{\varvec{c}}^{{{\text{APP}} - \tau J}} } \right)^{{\left( {ij} \right)}} \approx \frac{1}{h}{\text{Im}}\left[ {{{\varvec{\uptau}}}\left( {\widehat{{\varvec{F}}}^{{\left( {ij} \right)}} } \right)} \right] $$

Finally, the spatial algorithmic tangent moduli defined in ABAQUS are given as

40 $$ \left( {{\varvec{c}}^{{{\text{APP}} - {\text{MZ}} - {\text{J}}}} } \right)^{{\left( {ij} \right)}} \approx \frac{1}{Jh}{\text{Im}}\left[ {{{\varvec{\uptau}}}\left( {\widehat{{\varvec{F}}}^{{\left( {ij} \right)}} } \right)} \right] $$

Note that Eq. (40) is independent of the material model, and only an analytic equation for the Cauchy stress must be supplied in a UMAT.

In "Algorithmic spatial tangent moduli ", the Kirchhoff stress ${{\varvec{\uptau}}}_{n + 1}$ is obtained, so, the numerical spatial algorithmic tangent moduli required in UMAT is obtained easily using Eq. (40)

41 $$ \left( {{\varvec{c}}_{n + 1}^{{{\text{APP}} - {\text{MZ}} - {\text{J}}}} } \right)^{{\left( {ij} \right)}} \approx \frac{1}{{J_{n + 1} h}}{\text{Im}}\left[ {{{\varvec{\uptau}}}\left( {\widehat{{\varvec{F}}}_{n + 1}^{{\left( {ij} \right)}} } \right)} \right] $$

**Figure 2:**
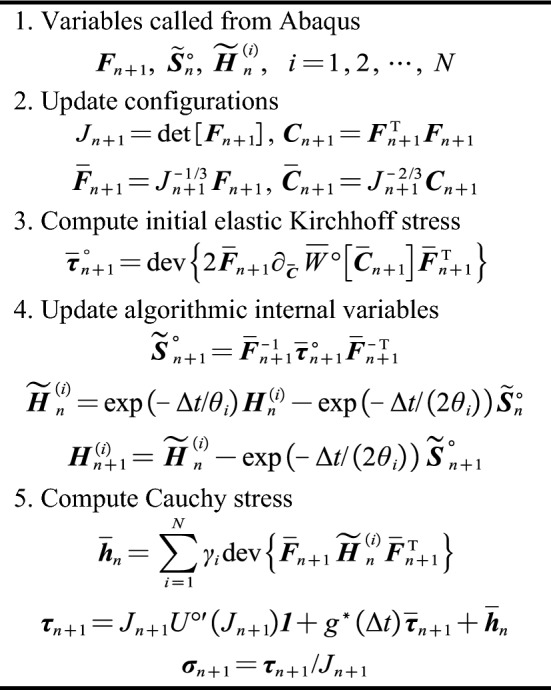
Recursive update implementation for Cauchy stress.

**Figure 3:**
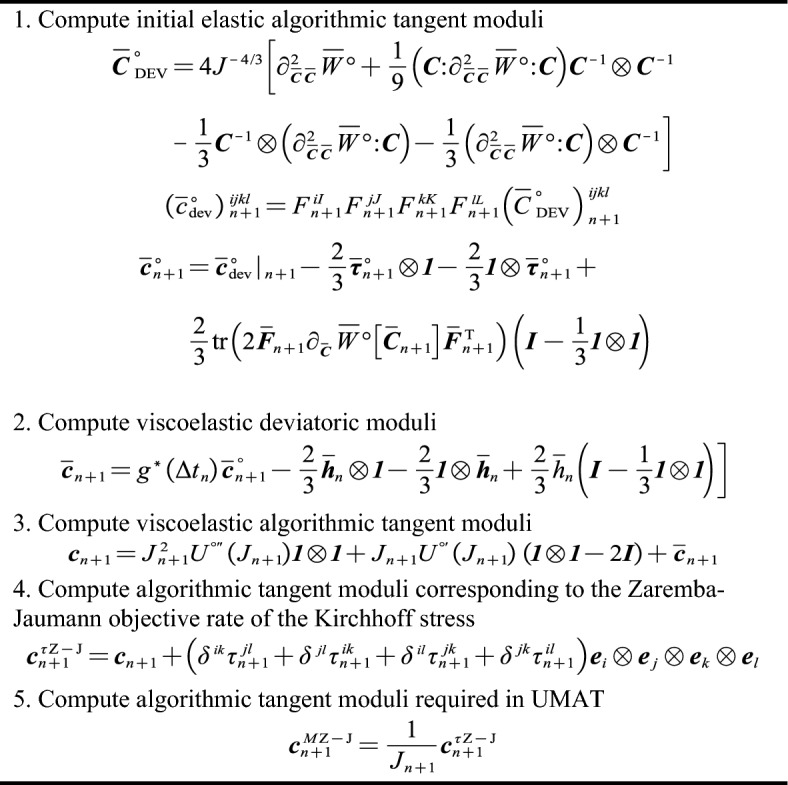
Recursive update implementation for analytical spatial algorithmic tangent moduli.

**Figure 4:**
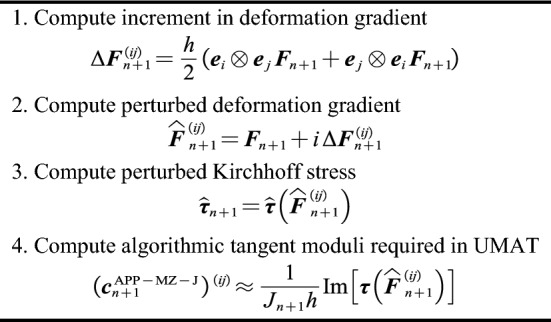
Recursive update implementation for numerical spatial algorithmic tangent moduli.

So far, closed equations form for the Cauchy stress and the spatial algorithmic tangent moduli and numerical spatial algorithmic tangent moduli have been formulated. The implementations of the algorithm for these variables are shown in Figs. 2, 3 and 4, respectively.

## Numerical testing

In this section, the numerical validation of stresses and tangent moduli of a class of nonlinear viscoelastic models for the two above-described methods is now analyzed. It is assumed that the model is incompressible, so, the initial deviatoric elastic strain energy $\overline{W}^{ \circ }$ becomes $W^{ \circ }$. The so-called Yeoh strain energy function is chosen as the $W^{ \circ }$, namely

42 $$ W^{ \circ } = C_{10} \left( {I_{1} - 3} \right) + C_{20} \left( {I_{1} - 3} \right)^{2} + C_{30} \left( {I_{1} - 3} \right)^{3} $$

Material parameters for the proposed model and perturbation parameter are given in Table 1^27^:

**Table 1:** Material parameters for the proposed model.

$\gamma_{\infty }$	$\gamma_{1}$	$\theta_{1}$(s)	$\gamma_{2}$	$\theta_{2}$(s)
16.8767	6.0408	0.0060	7.1988	0.0169

*C*_10_ [MPa]	*C*_20_ [MPa]	*C*_30_ [MPa]		
0.1588	3.0742	10.7827		

### Single cell test

**Figure 5:**
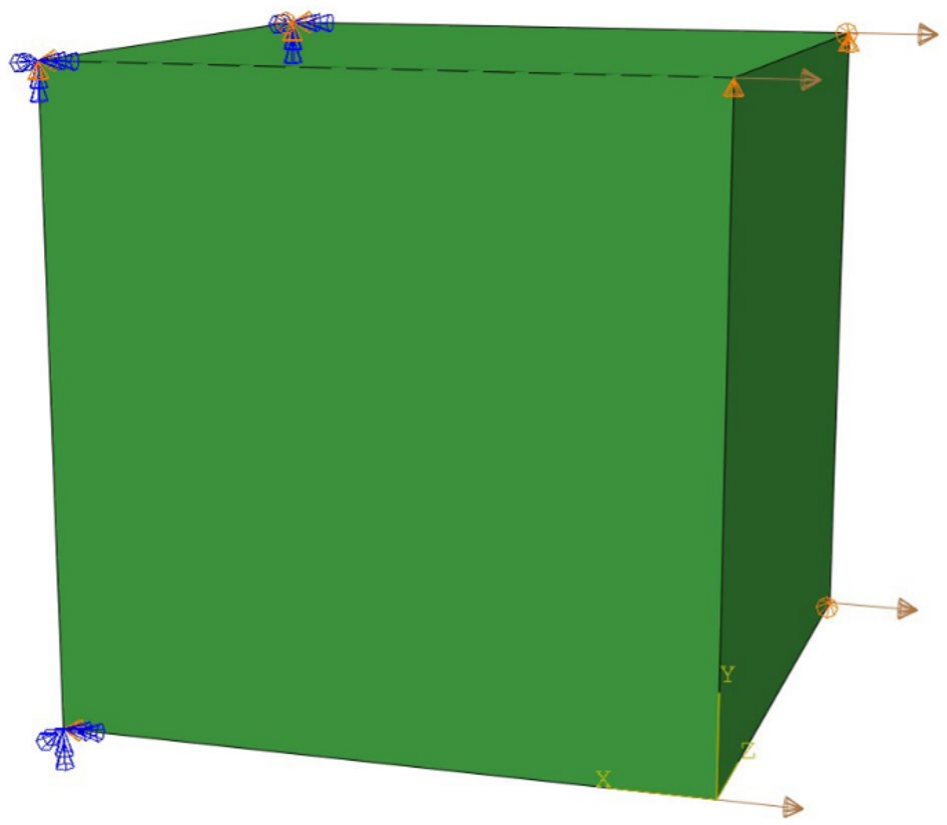
Boundary conditions of the single element.

In order to examine the relative error between the analytic tangent moduli and the numerical approximation tangent moduli evaluated using the CSDA scheme, a uniaxial deformation test is conducted on a single C3D8H element for both approaches. In ABAQUS, we need to initialize these two arrays: DFGRD0, DFGRD1, namely

$$ {\text{DFGRD}}0 = \left( {\begin{array}{*{20}c} 1 & 0 & 0 \\ 0 & 1 & 0 \\ 0 & 0 & 1 \\ \end{array} } \right) $$

$$ {\text{DFGRD}}1 = \left( {\begin{array}{*{20}c} 1 & 0 & 0 \\ 0 & 1 & 0 \\ 0 & 0 & 1 \\ \end{array} } \right) $$

and DTIME is 0.0001 s. The boundary conditions on the left side of the single element are: U1 = U2 = U3 = 0, UR1 = UR2 = UR3 = 0, and the boundary conditions on the right side of the single element are: V1 = 1 mm/s, as shown in Fig. 5.

**Figure 6:**
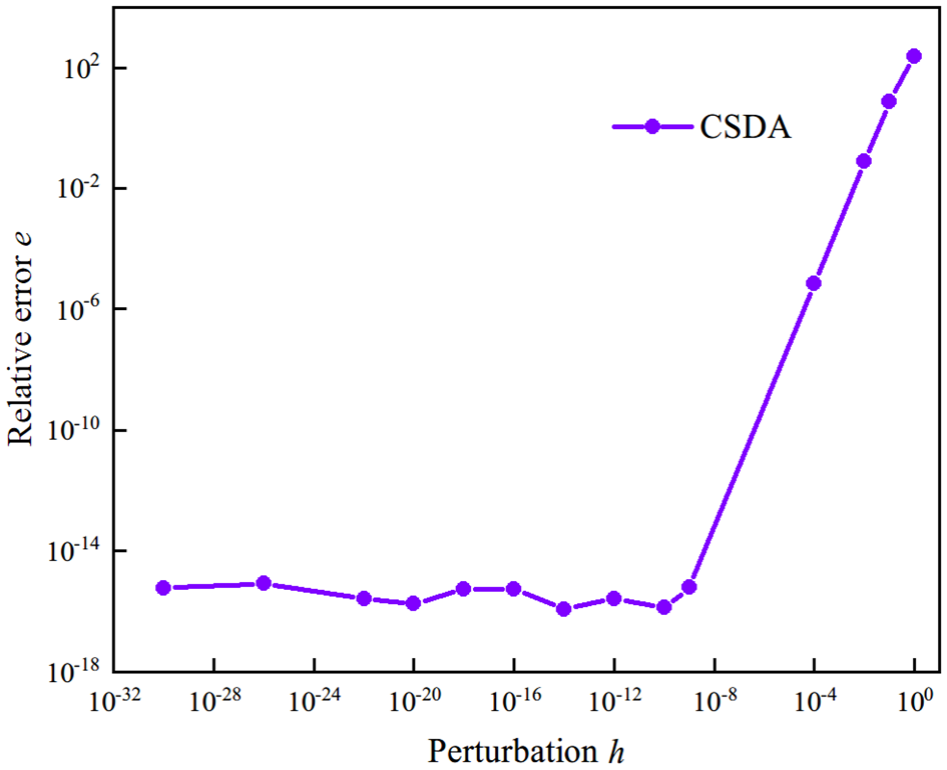
Relative error *e* of the numerical approximation tangent moduli.

To quantitatively compare the numerical approximation scheme with the analytical conclusion, the relative error *e* of these two methods with varying *h* is defined as

43 $$ e\left( h \right) = \sqrt {\frac{{\sum\limits_{i,j,k,l} {\left( {c_{ijkl}^{{{\text{analyt}}}} - c_{ijkl}^{{{\text{approx}}}} } \right)^{2} } }}{{\sum\limits_{i,j,k,l} {\left( {c_{ijkl}^{{{\text{analyt}}}} } \right)^{2} } }}} $$

where $c_{ijkl}^{{{\text{analyt}}}}$ denotes the component of analytic tangent moduli, $c_{ijkl}^{{{\text{approx}}}}$ denotes the component of numerical approximation tangent moduli. The perturbation parameter *h* varies from 1.0 × 10^–30^ to 1.0 × 10^0^ and the relative error *e* varies with perturbation parameter *h* is shown in Fig. 6. It is clearly that second order convergence is observed for CSDA numerical scheme (similar result is shown in ^20^). Increasing perturbation parameter *h* did not decrease the relative error *e* significantly when perturbation parameter *h* is smaller than 10^–9^. Different from FDM and CDM, In comparison to FDM and CDM, CSDA has a substantial advantage in that its rounding off mistakes are constrained by the lack of subtractive cancellation errors.

### Cook-type benchmarks

**Figure 7:**
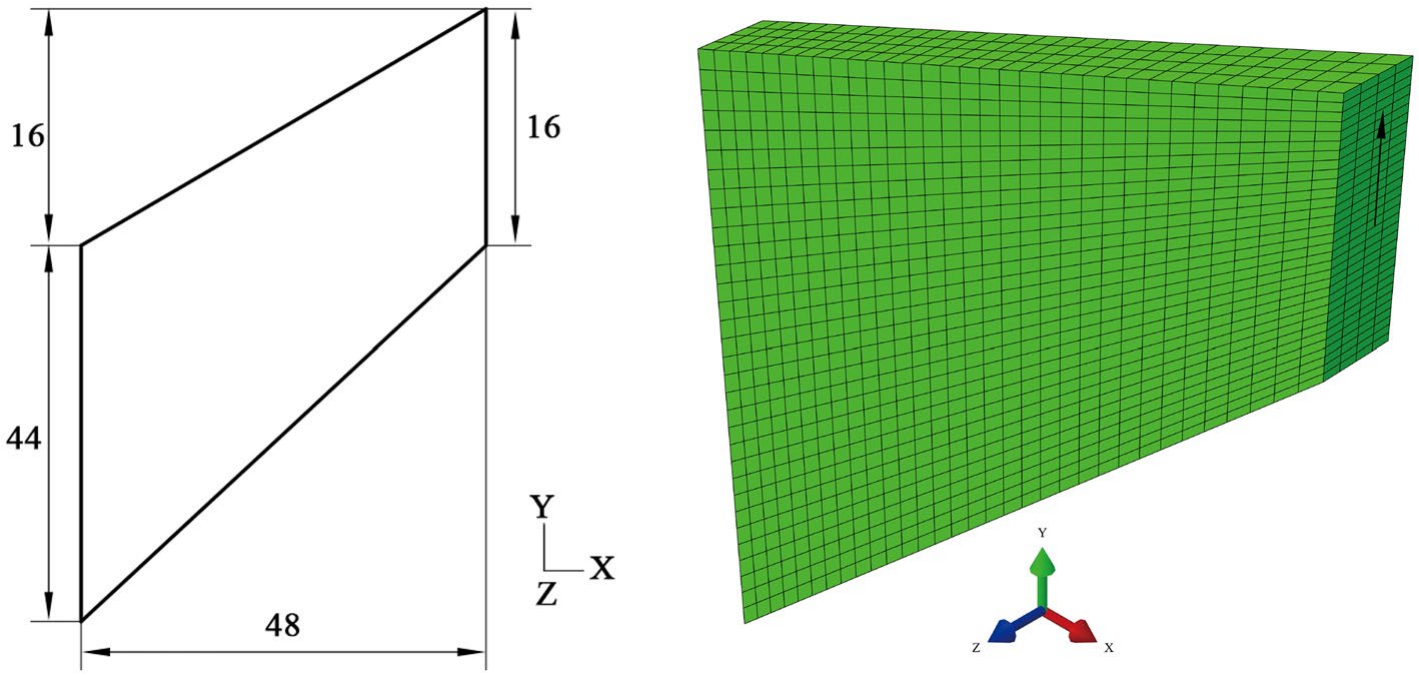
Initial geometry of the Cook’s membrane problem.

The so-called “Cook’s membrane problem” ^28^ is a standard inhomogeneous test, which combines shear and bending response with moderate distortion. As shown in Fig. 7. The left side of the section of Cook's membrane measures 44 mm, whereas the right side measures 16 mm. The top right corner is 16 mm higher than the top left corner. The distance between the two parallel sides measures 48 mm and the thickness of the Cook’s membrane along *Z* axis is 5 mm. the left-hand side of the Cook’s membrane is clamped (Namely, the conditions on the left-hand side of the Cook’s membrane are: U1 = U2 = U3 = UR1 = UR2 = UR3 = 0) and a 10 N concentrated shear force along *Y* axis is loaded on the center of the right section. Besides, symmetry boundary conditions (U3 = UR1 = UR2 = 0) are applied on the two *X*–*Y* plane faces. Due of the nearly incompressible nature of rubber, the model is meshed into 4097 elements and 8-node linear brick, hybrid with constant pressure (C3D8H) elements are used.

**Figure 8:**
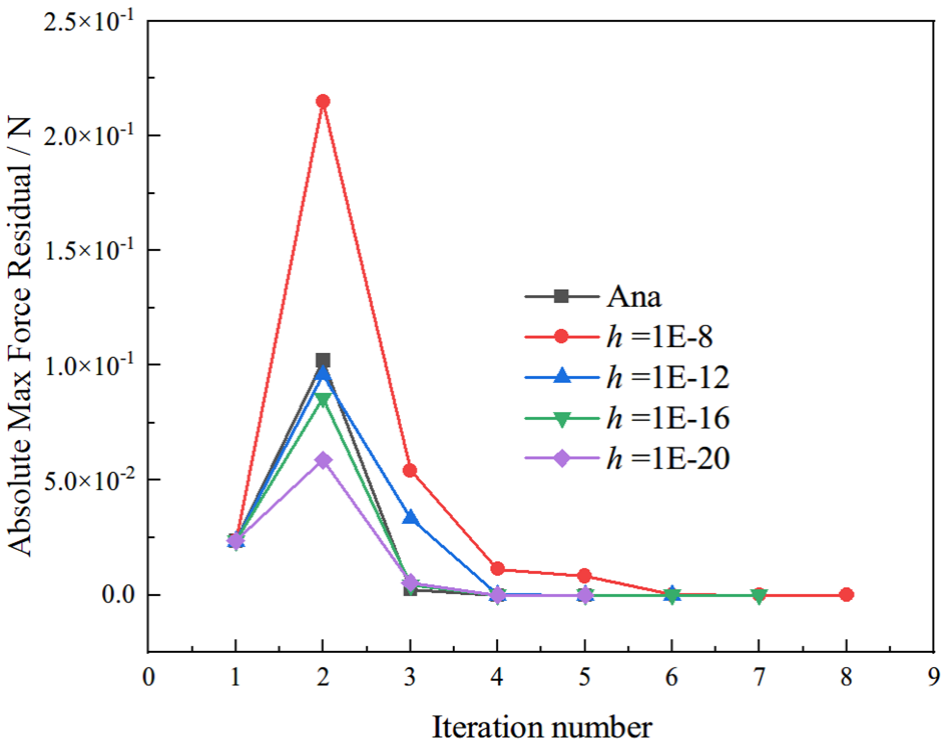
Absolute max force residuals vs iteration number with different perturbation parameter *h.*

**Figure 9:**
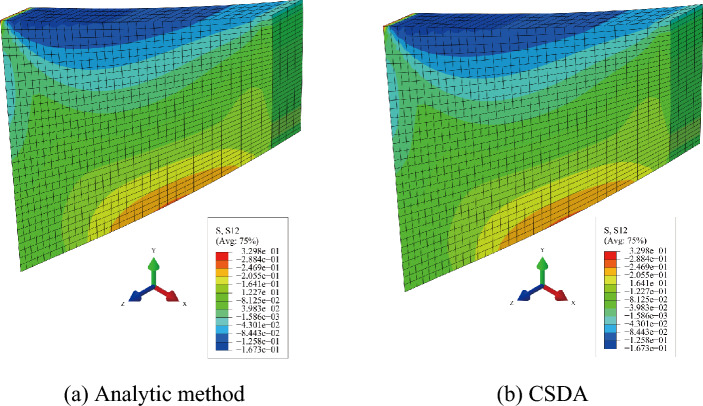
Distribution of Cauchy stresses $\sigma_{12}$ in the deformed configuration of the Cook-type problem resulting from different numerical schemes. (**a**) Analytic method, (**b**) CSDA.

To investigate the effect of perturbation parameter *h* on convergence bahavior, the maximum force residual at each iteration evaluating by analytic and approximation schemes are plotted for different perturbation parameter* h* shown in Fig. 8. As shown in Fig. 8, the maximum force residual at each iteration evaluated by the CSDA scheme becomes close to the analytical scheme with a decrease in perturbation parameter* h*. The resluts of the two schemes are very close when perturbation parameter* h* is equal to 1E−20. Figure 9a, b exhibit the Cauchy stress distributions *σ*_12_ in the last deformed configuration.

Next, the computing time of approximation scheme with different perturbation parameter *h* are investigated. The model is meshed into five levels of mesh refinement, namely 513, 4079, 14,400, 28,800 and 57,600 elements, and the perturbation parameter *h* is set to 1E−20. To assess computational efficiency of the two numerical schemes, we define so-called normalized total CPU time, namely

44 $$ t_{{{\text{Nor}}}} = \frac{{t_{{{\text{appro}}}} }}{{t_{{{\text{ana}}}} }} $$

**Figure 10:**
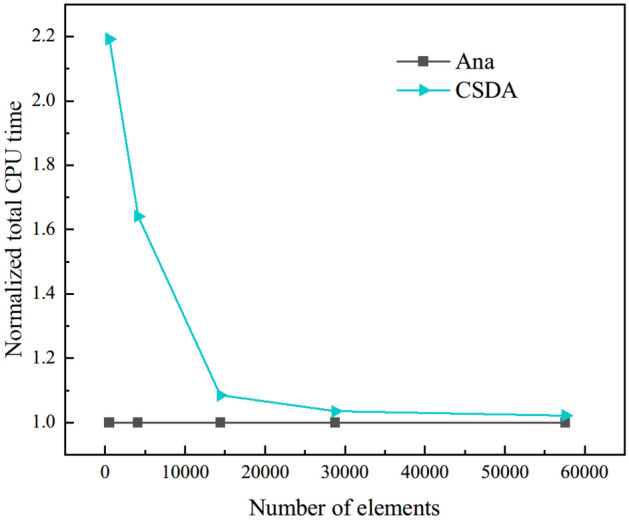
Comparison of computational costs of different numerical schemes.

The results are given in Fig. 10. As we expected, the normalized total CPU time decreases with the increase of mesh density. The normalized total CPU time of CSDA scheme is very close to one when the number of elements is equal to 57,600, because the amount of computation time associated with solving the system equations becomes increasingly dominant in comparison to the amount of time associated with assembling the system ^21^. Combing the discussion in “Single cell test” and "Finite element modeling of uniaxial tension", the optimal perturbation parameter *h* in our research is 1E−20. We select *h* = 1E−20 as a given parameter for further analyses in the following section.

### Finite element modeling of uniaxial tension

**Figure 11:**
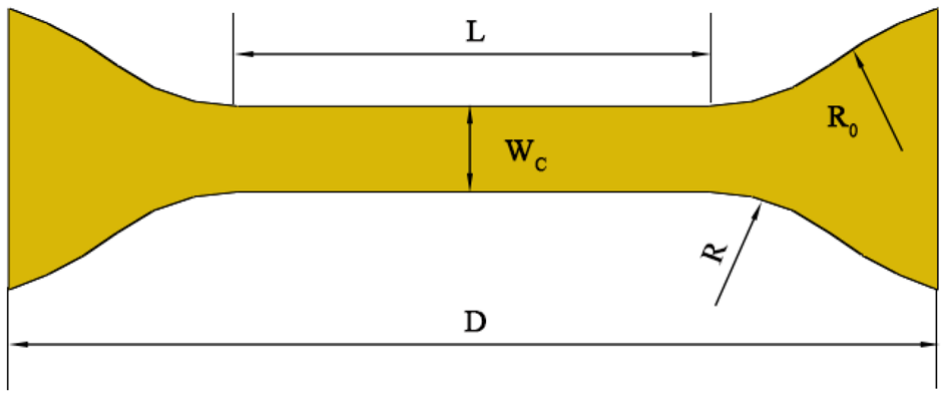
Geometry of dumbbell specimen.

**Figure 12:**
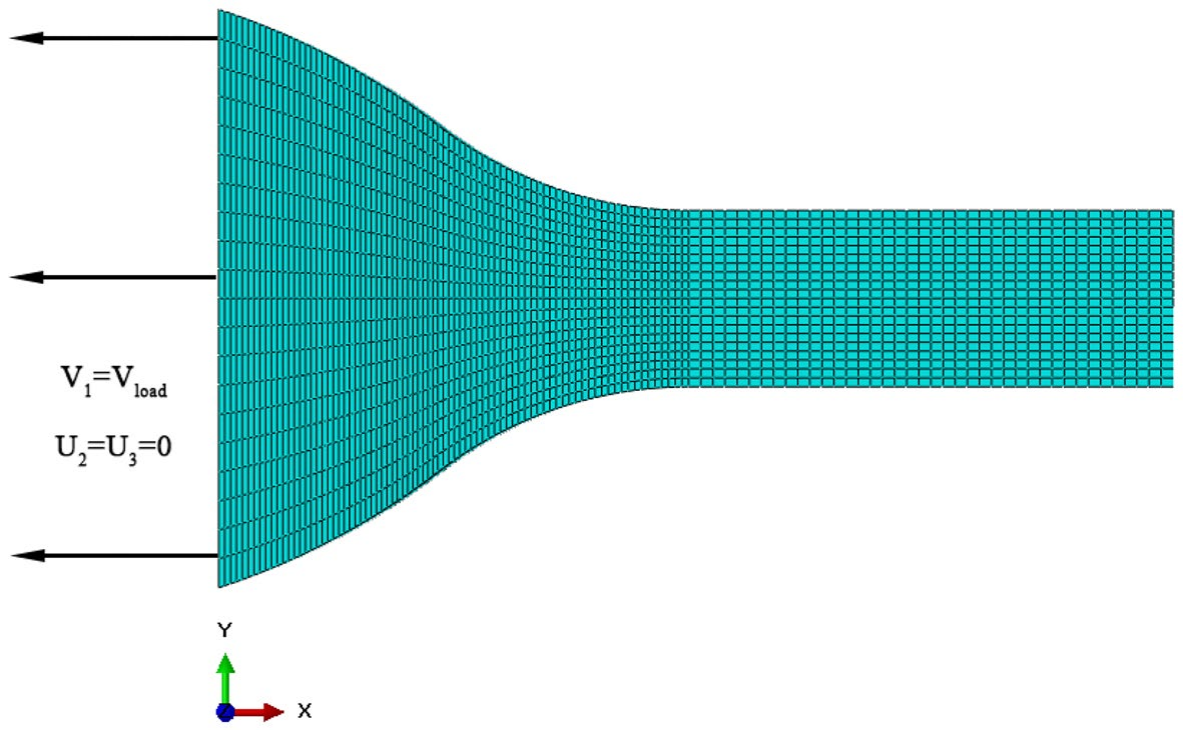
Boundary conditions and meshes for dumbbell specimen.

Figure 11 depicts the finite element model of unaxial tension utilized in this study^29^. The following are the dimensions of the dumbbell specimen: D = 50 mm, W_C_ = 6 mm, L = 20 mm, R = 10 mm, R_0_ = 25 mm, and thickness T = 1 mm. Initially, the finite element simulation model is stress-free. The boundary conditions of dumbbell-specimen are illustrated in Fig. 12 :$u_{2} = u_{3} = 0$, $v_{1} = v_{{{\text{loading}}}}$, where *u*_*i*_ denotes the component of displacement vecotr and *v*_load_ is the loading velocity. The dumbbell-specimen has been meshed into a total of 6784 C3D8H elements.

**Figure 13:**
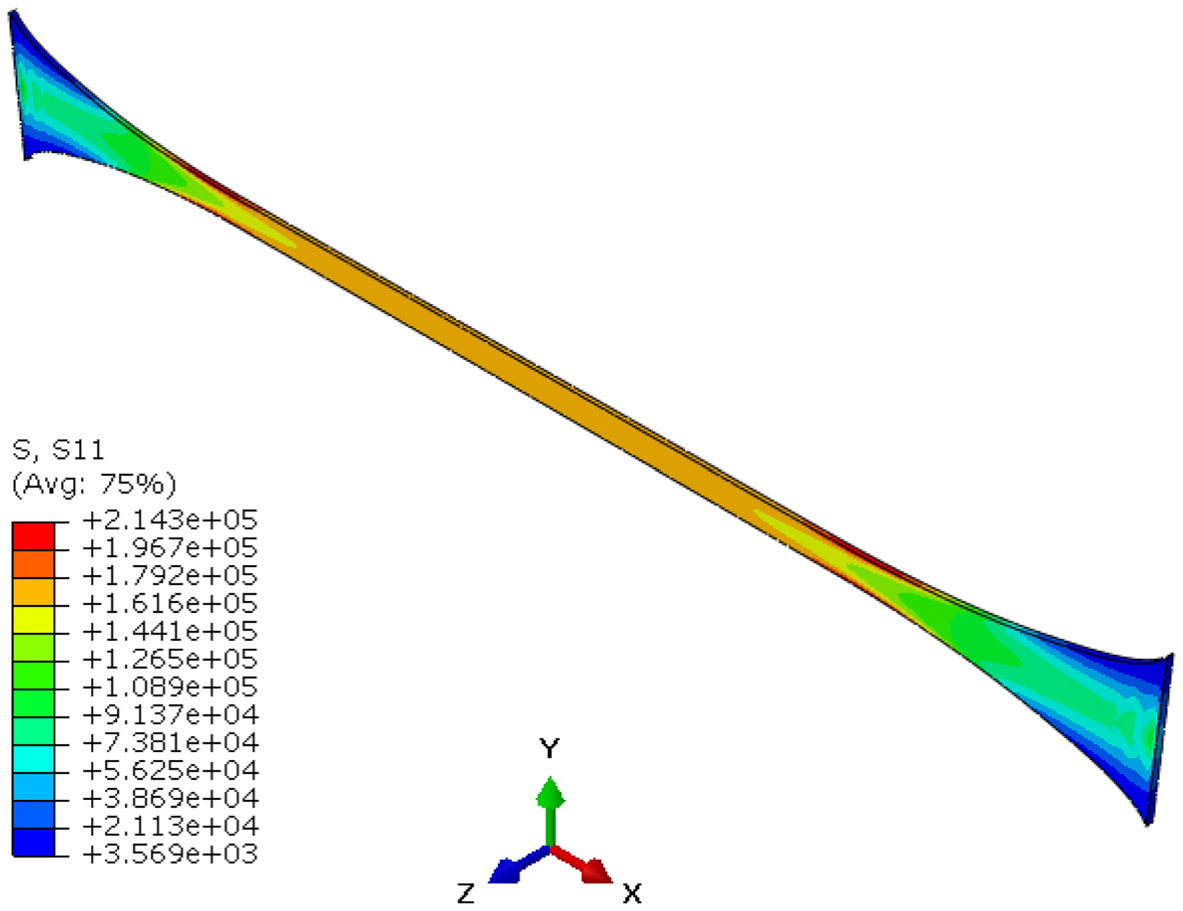
Cauchy stress *σ*_11_ contour of constant uniaxial tension at *t* = 10 s.

**Figure 14:**
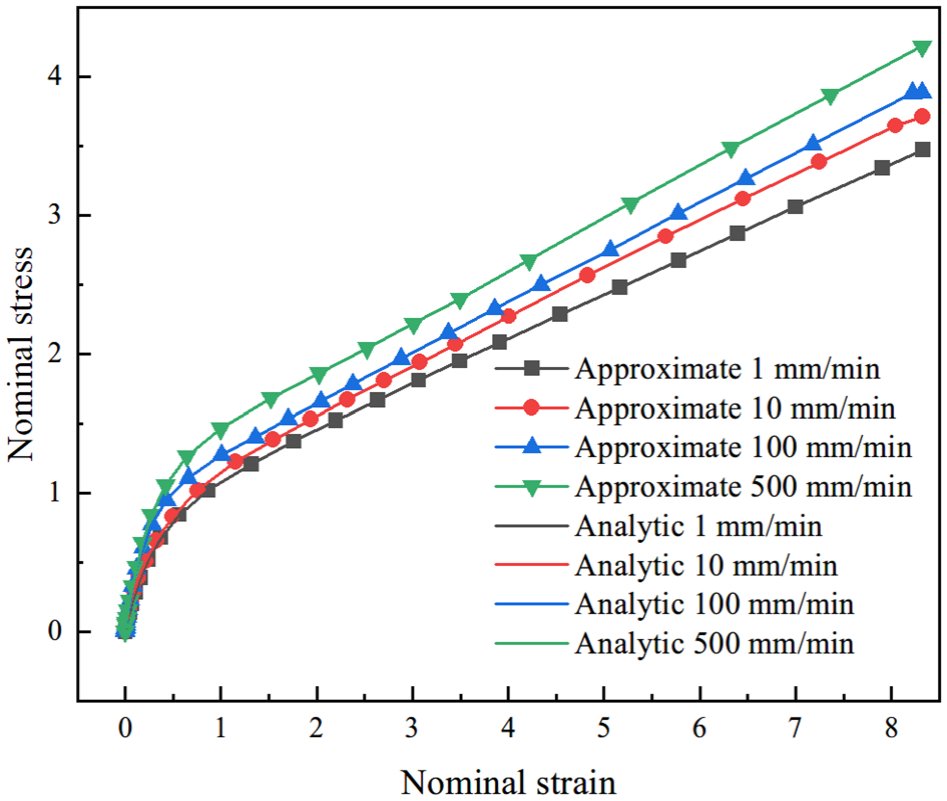
Comparison of uniaxial constant tension for analytic solution and CSDA method.

Cauchy stress contour along the loading direction at *t* = 10 s is shown in Fig. 13. The Cauchy stress and nominal strain at integration point 1 of element 2264 (coordinate origin of model) are extracted and graphed (shown in Fig. 14). As shown in Fig. 14, the analytical method of uniaxial constant tension are compared with results of CSDA scheme. It is found that the nominal stress–strain curves of the two different calculation methods coincide well, which reveals satisfactory performance of the CSDA method presented in the preceding section.

**Figure 15:**
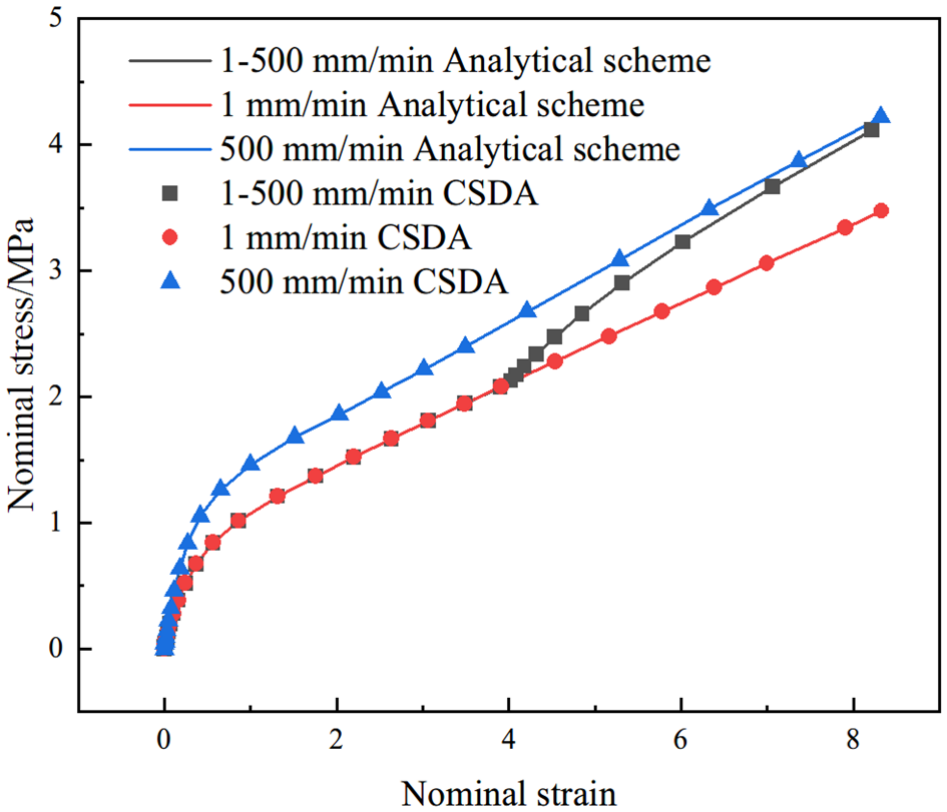
Comparison of uniaxial variable tension for analytical scheme and CSDA method.

**Figure 16:**
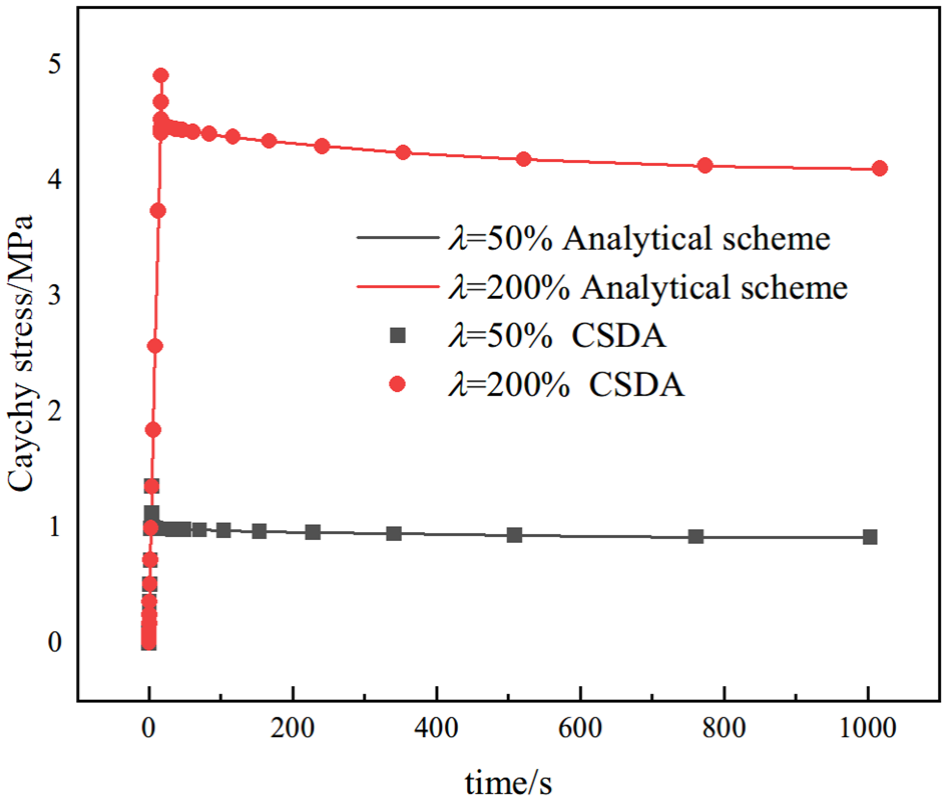
Comparison of stress relaxation for analytical scheme and CSDA method.

In order to further validate the CSDA method, Two other loading cases were simulated: (1) uniaxial variable tension, and (2) stress relaxation. As shown in Figs. 15and Fig. 16, the good agreement between the analytical scheme (line) and CSDA method (scatter) are observed, which further prove the correctness of the numerical algorithm (CSDA).

## Conclusions

In this work, we have proposed a numerical approximate algorithm for algorithmic tangent moduli for a class of nonlinear viscoelastic model. The analytical and numerical approximate tangent moduli algorithm approximate algorithms have been implemented into commercial finite element software ABAQUS/Implicit via a user subroutine. Computational performance using analytical and CSDA approaches has been tested in three different numerical tests. Each of these numerical methods is examined closely in terms of its convergence and computing efficiency. The simulation results show that the CSDA method is mathematically concise, which will greatly help to implement complex constitutive model in the finite element software.

## Data Availability

Data available on request from the authors: If there are any questions about this paper, Please contact Xinggui Fan, Email: 317101020020@njust.edu.cn.
